# Investigating Adiposity-Related Metabolic Health Phenotypes in Patients with Hidradenitis Suppurativa: A Cross-Sectional Study

**DOI:** 10.3390/jcm12144847

**Published:** 2023-07-23

**Authors:** Dillon Mintoff, Rachel Agius, Stephen Fava, Nikolai P. Pace

**Affiliations:** 1 Department of Pathology, Faculty of Medicine and Surgery, University of Malta, MSD2080 Msida, Malta; dillon.mintoff@gov.mt; 2 Department of Dermatology, Mater Dei Hospital, MSD2090 Msida, Malta; 3 Department of Medicine, Faculty of Medicine and Surgery, University of Malta, MSD2080 Msida, Malta; rachel.agius@gov.mt (R.A.); stephen.fava@gov.mt (S.F.); 4 Department of Medicine, Mater Dei Hospital, MSD2090 Msida, Malta; 5 Department of Anatomy, Faculty of Medicine and Surgery, University of Malta, MSD2080 Msida, Malta; 6 Centre for Molecular Medicine and Biobanking, University of Malta, MSD2080 Msida, Malta

**Keywords:** hidradenitis suppurativa, obesity, phenotypes, metabolic health

## Abstract

Background: Obesity and hidradenitis suppurativa (HS) are related through meta-inflammation and are both associated with increased cardiometabolic risk. Notwithstanding, cardiometabolic pathology is not uniform in obesity and a subset of individuals with excess adiposity exhibit a healthy metabolic profile. Whilst the incidence of cardiometabolic endpoints and transitions across different adiposity-related body composition phenotypes within several populations and across different ethnicities have been investigated, data regarding metabolic health (MetH) and body composition phenotypes in individuals with HS are lacking. The objective of this study was to evaluate the relationship between different body composition phenotypes in individuals with HS. Methods: This was a cross-sectional study of 632 individuals with and without HS from a population with a high prevalence of both obesity and HS. A total of four body composition phenotypes were generated based on BMI and metabolic status (defined using either the metabolic syndrome definition or the homeostasis model of insulin resistance (HOMA-IR)): metabolically healthy overweight/obese (MHOWOB), metabolically unhealthy overweight/obese (MUOWOB), metabolically healthy normal weight (MHNW), and metabolically unhealthy normal weight (MUNW). Results: Generally, subjects with HS exhibited a worse metabolic profile with higher levels of indices of central adiposity measures (including Visceral Adiposity Index and waist circumference), systolic blood pressure and markers of insulin resistance, as well as a higher prevalence of the metabolic syndrome. Moreover, when sub-stratified into the different body composition phenotypes, individuals with HS typically also demonstrated adverse metabolic characteristics relative to controls matched for both adiposity and metabolic health, particularly in the normal weight category and despite being classified as metabolically healthy. Being metabolically unhealthy in addition to being overweight/obese increases an individual’s risk of HS. Conclusions: Metabolic risk-assessment should be prioritized in the clinical management of individuals with HS even in those who are lean. Patients attending HS clinics provide a valuable opportunity for targeted cardiovascular risk reduction with respect to the management of both obesity and metabolic health.

## 1. Introduction

Hidradenitis suppurativa (HS) is a chronic, inflammatory disease of the pilosebaceous unit (PSU). Lifestyle, environmental and genetic factors drive hyperkeratinisation and auto-inflammation at the PSU. The management of HS is directed towards abating these processes through both medical and surgical interventions [1]. Individuals with HS are typically obese, smokers and are at a higher risk of developing the metabolic syndrome (MetS) (Odds Ratio 2.66, 95%CI: 1.90–3.72) [2]. HS and obesity share common pathophysiological underpinnings, characterized by systemic, low-grade chronic inflammation and a dysregulated adipokine profile [3,4]. Relevantly, metabolic and immune pathways are conserved and interdependent [5]. Moreover, inflammatory alterations in hypothalamic neuroendocrine pathways regulating satiety and energy expenditure are implicated in the onset of obesity and metabolic pathology [6].

Obesity (body mass index [BMI] of ≥30 mg/kg^2^ [ICD-11: 5B81]) predisposes to insulin resistance and cardiometabolic events. However, obesity is a heterogenous condition and the occurrence of cardiometabolic pathology is not uniform across individuals with excess adiposity [7]. A subset of individuals with obesity exhibit a healthy metabolic profile (referred to as metabolically healthy obese [MHO]) [8]. Conversely, some normal weight individuals display metabolic abnormalities typically associated with excess adiposity (referred to as metabolically unhealthy normal weight [MUHNW]) [9]. While several studies have investigated the incidence of cardiometabolic endpoints and transitions across adiposity-related body composition phenotypes [10,11], data regarding metabolic health (MetH) and body composition phenotypes in individuals with HS are lacking. The definition of MetH is heterogenous with several proposed diagnostic criteria based on combinations of anthropometric and biochemical parameters [12].

Given the established association and high prevalence of both HS and major adverse outcomes (such as type 2 diabetes mellitus (T2DM), cardiovascular (CV) disease and all-cause mortality) the appropriate characterization of MetH in HS should be prioritized [13,14]. Based on clinical evidence, stratification of comorbid metabolic risk in HS is recommended as part of comprehensive clinical care strategies, and supported by preliminary evidence showing that metformin yields beneficial effects in HS patients [15,16]. 

Against this background, this study sought to evaluate adiposity-related MetH phenotypes in HS by carrying out a cross-sectional analysis of a cohort of individuals with HS of Maltese-Caucasian ethnicity, a Mediterranean island population having a high prevalence of both obesity and HS [17]. Additionally, we aimed to evaluate links between HS severity and disease phenotypes across MetH definitions.

## 2. Materials and Methods

### 2.1. Ethical Approval 

This study was carried out in accordance with the Declaration of Helsinki and approved by the institutional ethics review board of the University of Malta (UREC MD 06/2016 and UREC 827_05042021). Written consent was obtained from all patients participating in this study. The study followed the Strengthening the Reporting Studies in Epidemiology (STROBE) guidelines. 

### 2.2. Study Population

Eligibility for enrolment of adult HS patients was conducted by a dermatologist leading the national HS clinic (Dermatology Department at Mater Dei Hospital, Malta). The department is the exclusive public dermatology referral centre in the Maltese archipelago (550,000 inhabitants). The Dessau Definition of HS was adopted [18]. HS severity was assessed using Hurley staging [19] and disease phenotype was based on definitions proposed by Canoui-Poitrine (LC) [20]. Patient recruitment was carried out from January to December 2022. 

The control population comprised 521 adults of Maltese-Caucasian ethnicity, recruited through a method of convenience sampling as part of another cross-sectional study concerning the prevalence of the different body composition phenotypes within a nationally representative sample of middle-aged non-institutionalized Maltese-Caucasians. Details of the research design and study protocol have been described elsewhere [21]. Subjects with a history of type 1 diabetes mellitus, underlying genetic or endocrine causes of overweight or underweight (apart from controlled thyroid disorders), terminal illness or active malignancy, pregnant females and individuals who could not give their voluntary consent were excluded. Patients suspected or diagnosed with HS were excluded from the control group.

Baseline demographic and clinical parameters were recorded at enrolment for both patient groups. Anthropometric measurements were taken on patients wearing light-clothing using calibrated instruments. Normal weight, overweight and obesity were defined as BMI of 18.5–24.9 kg/m^2^; 25.0–29.9 kg/m^2^ and ≥30 kg/m^2^ respectively.

Bloodletting was carried out after an overnight fast. Hematologic and metabolic parameters were determined using standard hospital biochemical analysers. The homeostasis model assessment (HOMA) was used to evaluate insulin resistance (IR) [22]. The cut-off value for HOMA-IR to define insulin resistance was set at ≥2.5 based on other studies linking HOMA-IR to all-cause mortality [23,24].

### 2.3. Defining Metabolic Health and Adiposity Body Composition Phenotypes

Body size phenotypes were generated based on the combined consideration of each participants’ BMI category and MetH status. Two separate definitions of MetH were applied to cross-classify study participants:

(a) Definition 1: Participants were considered to be metabolically healthy if they had one or none of the following NCEP-ATPIII components:
  - elevated triglycerides (TG) (≥1.7 mmol/L) or treatment with lipid-lowering drugs,
  - elevated systolic blood pressure (SBP) (≥130 mmHg) or diastolic blood pressure (DBP) (≥85 mmHg) or treatment with anti-hypertensive drugs,
  - elevated fasting glucose (≥5.6 mmol/L) or on antihyperglycemic agents and low High Density Lipoprotein-C (HDL-C) (<1.03 mmol/L in males and <1.29 mmol/L in females) or on treatment aimed to increase HDL-C.

This definition excludes WC in view of its collinearity with BMI [25].

(b) Definition 2: Absence of insulin resistance as denoted by a HOMA-IR value of <2.5 [24,26].

Overweight and obese subjects were considered as a single aggregate category thus generating four body composition phenotypes:

- Metabolically healthy normal weight (MHNW),
- Metabolically unhealthy normal weight (MUHNW),
- Metabolically healthy overweight/obese (MHOWOB) and
- Metabolically unhealthy overweight/obese (MUHOWOB).

Using the HOMA-IR definition of MetH, only a single HS patient was classified as MUHNW, and this category was eliminated from downstream analysis. 

A diagnosis of MetS was made in individuals fulfilling ≥3 of the NCEP-ATPIII parameters.

Visceral adiposity index (VAI) and atherogenic index of plasma (AIP) were calculated from clinical and biochemical parameters [27,28]. 

### 2.4. Statistical Analysis

The normality of continuous variables was assessed by the Shapiro–Wilk and Kolmogorov–Smirnoff tests. All continuous parameters exhibited a skewed non-normal distribution, and non-parametric statistics with medians and interquartile ranges are presented. Categorical variables are presented as percentages and the χ^2^ test was applied to compare dichotomous outcomes. The Kruskal–Wallis/Mann–Whitney U test were used for comparison of quantitative variables.

Binary logistic regression, adjusted for age, gender and smoking status was used to evaluate the association between (a) MetS, (b) its constituent individual components, (c) overweight/obesity, (d) adiposity body composition phenotypes as separate independent predictors and HS risk as the dependent response variable. MetS components were considered as binary categorical variables as their definitions incorporate drug use for the management of hypertension, dyslipidaemia, and hyperglycaemia. Statistical analysis was performed using IBM SPSS v26 (Chicago, IL, USA) and R v.3.4.2. Odds ratios and 95% CI are reported, and a *p* value of <0.05 was considered statistically significant.

## 3. Results

### 3.1. Population Characteristics

A total of 632 individuals were included in this analysis, of which 111 (17.6%) were diagnosed with HS. In total, 521 individuals served as the control reference population. The salient characteristics of study cohorts at baseline are presented in Table 1.

Despite age and sex difference between case and control groups, individuals with HS exhibited a less favourable metabolic profile overall. While subjects with HS were younger, they were more likely to smoke, have a higher BMI, WC and SBP. With respect to biochemical parameters, significantly higher HOMA-IR, VAI, AIP, NLR, and red-cell distribution width (RDW) and significantly lower HDL-C levels were observed in the HS group. Furthermore, a higher proportion of individuals with HS fulfilled the NCEP-ATP III criteria for MetS (20.3 vs. 32.4%, *p* < 0.001).

A comparison of clinical and biochemical characteristics in individuals with HS vs. controls stratified by body composition phenotype is presented in Table 2 and Table 3. Across both definitions of MetH, individuals with HS falling within the MHOWOB and MUHOWOB category were more likely to be smokers, younger, hypertensive, and have a greater WC and NLR compared to controls. Moreover, despite being classified as ‘healthy’, individuals with HS within the MHNW category exhibited significantly higher WC, TG and HOMA-IR relative to controls.

### 3.2. Relationship between the Different Body Composition Phenotypes and HS

**Figure 1:**
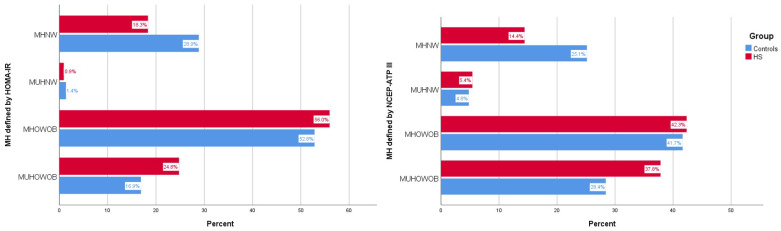
Prevalence of the four body composition phenotypes using different definitions of metabolic health: HOMA-IR (left panel) and NCEP-ATPIII (right panel). MHNW: metabolically healthy normal weight, MUHNW: metabolically unhealthy normal weight, MHOWOB: metabolically healthy overweight-obese, MUHOWOB: metabolically unhealthy overweight obese, MH: metabolic health.

The association between the body composition phenotypes and HS was next evaluated. A significant difference in the distribution of the four adiposity phenotypes between HS and controls was observed when MetH was defined by either HOMA-IR (χ^2^ = 7.1, *p* = 0.005) or NCEP-ATP III (χ^2^ = 7.31, *p* = 0.007) criteria (Figure 1). Notably, a significantly higher proportion of individuals with HS were classified as MUHOWOB by both definitions but a lower proportion were classified as MHNW. 

Binary logistic regression analysis was used to evaluate the association between (1) MetS, (2) its constituent individual components and (3) overweight/obesity based on BMI cut-offs and HS risk as the response variable. In age and sex-adjusted models, overweight/obesity, the MetS, FPG and hypertension were significantly associated with HS risk in the study population. In age-adjusted and sex-stratified analysis, increased WC was an HS risk factor in both sexes, while low HDL-C levels increased HS risk in females but not in males. 

**Figure 2:**
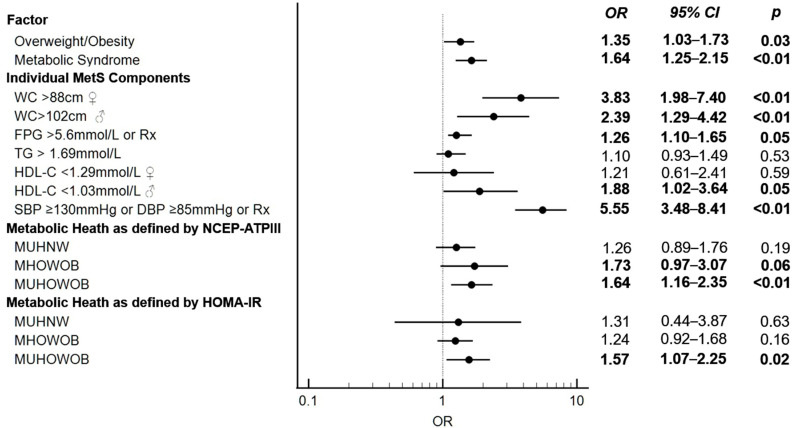
Forest plot summarizing HS risk for individual components of the metabolic syndrome and body composition phenotypes using two definitions of metabolic health. WC: Waist circumference, FPG: Fasting plasma glucose, TG: triglycerides, HDL: high-density lipoprotein, SBP: Systolic blood pressure, HOMA-IR: Homeostatic Model Assessment for Insulin Resistance, OR: Odds ratio.

We next evaluated the association between the four body composition phenotypes and HS risk. In age- and sex- adjusted models, the MUHOWOB category was associated with a significantly higher risk of HS, with a similar magnitude of effect size across both definitions of MetH (Figure 2).

### 3.3. Relationship between HS Hurley Severity/LC Phenotype and BMI/HOMA-IR Parameters

**Figure 3:**
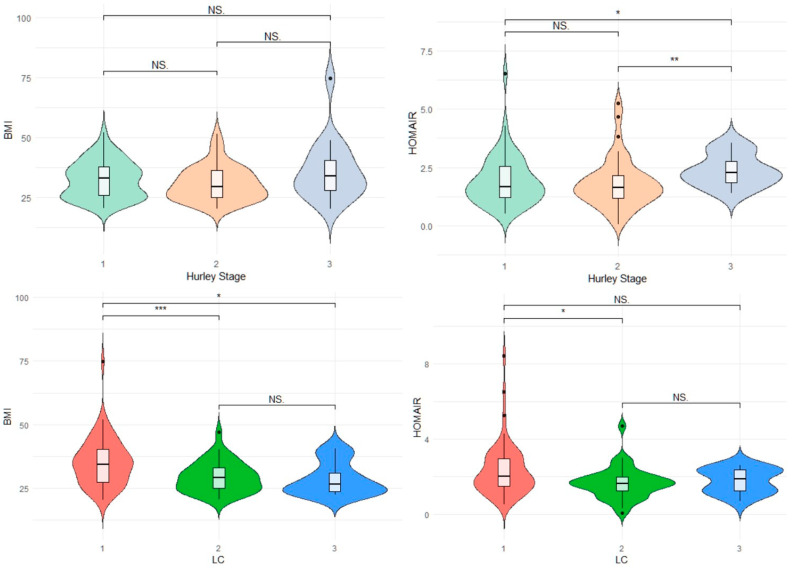
Violin plots illustrating relationship between HS Hurley Severity/LC phenotype and BMI/HOMA-IR parameters * *p* ≤ 0.05 ** *p* ≤ 0.01 ** *p* ≤ 0.001 BMI: Body mass index in kg/m. NS: Non significant, LC: Latent Class Phenotype.

We next evaluated adiposity-related phenotypes in the HS group across categories defined by LC and Hurley severity. Of the 111 individuals with HS, 47.7% (n = 53), 36.9% (n = 41) and 15.3% (n = 17) were classified as suffering from Hurley Stage I, II and III disease, respectively. With regards to the LC phenotype, 54.9% (n = 61), 33.3% (n = 37), 11.7% (n = 13) of patients were categorized as having LC class 1, 2 and 3 disease, respectively. No significant differences in gender proportions Hurley categories were present. Patients in each Hurley severity stage had comparable BMI, however patients with Hurley 3 disease severity had a higher HOMA-IR when compared to those suffering from Hurley 1 (*p* = 0.029) and Hurley 2 (*p* ≤ 0.01) disease severity (Figure 3). 

HS patients manifesting the LC1 phenotype had a higher BMI than patients with LC2 (*p* ≤ 0.001) and LC3 (*p* = 0.013) phenotype. A significantly higher HOMA-IR was present in LC1 HS phenotype (*p* = 0.012). (Figure 3)

No significant difference in the distribution of body composition phenotypes between Hurley Severity Stages was identified. This was consistent across the NCEP-ATPIII (χ^2^ = 3.81, *p* = 0.435) and HOMA-IR (χ^2^ = 3.58, *p* = 0.495) definitions of MetH.

**Figure 4:**
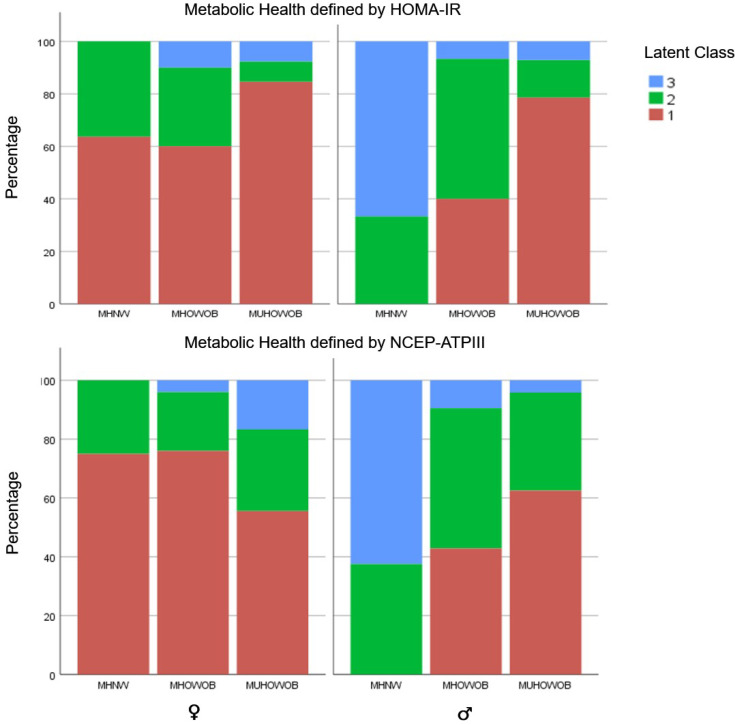
Gender-stratified analysis of body composition phenotypes in Latent class phenotype/metabolic health (as defined by HOMA-IR and NCEP-ATPIII).

In gender-stratified analysis, a significant difference in body composition phenotypes was observed across LC using either definition of MetH (χ^2^ = 29.3, *p* < 0.001). HS cases classified as MUHOWOB had a significant predilection for LC1 (axillary/breast disease) in both genders, while MHNW HS cases were predominantly LC1 in females and LC3 (gluteal) in males (Figure 4).

## 4. Discussion 

In this study, we apply two established definitions of MetH to explore the characteristics of the different body composition phenotypes in an HS cohort. These data reveal important metabolic implications for patients with HS. Despite being younger, the HS group exhibited an unfavourable metabolic profile characterised by higher adiposity, systolic hypertension, insulin resistance, a higher prevalence of the MetS and elevated inflammatory burden. When sub-stratified into different body composition phenotypes, HS patients generally demonstrated a worse metabolic milieu relative to controls matched for both adiposity and MetH, particularly in the normal weight category. Furthermore, we also show that the MUHOWOB category is a predictor of HS risk across both definitions of MetH. 

Epidemiological studies show that overweight/obesity status unstratified by MetH status is a significant HS risk factor. While our findings support this association, this study goes on to demonstrate that the presence of an unhealthy metabolic profile in addition to being overweight or obese increases an individual’s risk of developing HS. This has direct clinical implications and reinforces the importance of intensive metabolic risk stratification in HS patients. 

We also demonstrate that increased insulin resistance as defined by HOMA-IR is associated with worse HS severity, although BMI did not differ across categories of disease severity. This analysis also suggests that gender-specific patterns of anatomic site involvement in HS defined by the LC system are related to adiposity phenotypes. Specifically, the MUHOWOB category had a higher predilection for LC1 HS in both genders, while HS patients classified as MHNW exhibit a gender dimorphic pattern in LC classification. 

The association between HS, obesity and cardiometabolic risk has robust physiological and epidemiological underpinnings. Physiologically, chronic systemic inflammation underlies the pathogenesis of both HS and atherosclerotic cardiovascular disease. The proinflammatory state leads to dysregulated insulin metabolic signalling within insulin sensitive tissues as well as pancreatic β cell dysfunction, insulinopenia and endothelial dysfunction [29,30,31,32,33]. This thus represents an important mechanism linking obesity and HS to the onset of cardiometabolic disease. Recently, increased expression of Hypoxia inducible Factor-1α was identified in serum and lesional skin of individuals with HS, a vital transcription factor for glycolysis [34].

An elevated risk of adverse cardiometabolic outcomes, including ischaemic heart disease and cerebrovascular disease–independent of age, sex and smoking status -has been reported in HS [14,35]. Multiple studies explored the prevalence of MetS in HS. Our reported prevalence (32.4%) is lower than that reported internationally (40%) [36], although comparison is limited by differences in study characteristics, patient ascertainment criteria and populations studied. Stratification of HS by underlying metabolic risk factors holds considerable potential for targeted lifestyle and pharmacological intervention. Precision phenotyping of complex metabolic disease based on mechanisms is proven superior to traditional clinical classifications, as it better identifies patients at risk of complications and can guide therapeutic choices [37]. A higher prevalence of subclinical atherosclerosis as assessed by carotid artery intima thickness (cIMT), independent of classical cardiovascular risk factors, has also been reported in HS [38,39]. Longitudinal studies show that adalimumab confers beneficial cardiovascular risk with a reduction in cIMT in a subset of HS patients exhibiting classical metabolic risk factors [40]. This further reinforces the need for stratified cardiovascular risk management in HS, as cIMT non-responders to adalimumab exhibited a different HS phenotype. Our analysis also lends support for the importance of incorporating MetH over and above sole use of the BMI in HS risk stratification. Importantly, HS patients might constitute a valuable population for targeted cardiovascular risk reduction as they are usually younger and less likely to visit general physicians, although more likely to regularly consult dermatologists [35].

This study is strengthened by the use of a large, ethnically matched reference population unselected for HS recruited through convenience sampling from the general population which aids generalisation of results. The use of a well-phenotyped cohort of middle-aged adults within a narrow age range overcomes issues related to survival bias and sarcopenia in older adults. 

Some limitations merit consideration. Primarily, the cross-sectional study design limits evaluation of the direction of causality and considers metabolic factors as exposure variables driving HS. Both excess adiposity and HS share a systemic proinflammatory state and thus reverse causation is plausible. This study did not incorporate data on diet, proinflammatory cytokines, measurements of visceral adiposity or cardiorespiratory fitness. BMI was used as an index of obesity measurement and thus, it could have misclassified individuals with short stature or increased muscle mass. The control population was not matched for age, gender, and smoking status, although these factors were considered as confounders in statistical analysis. We consider adiposity and MetH as static factors and thus dynamic transitions across metabolism-weight phenotypes could not be assessed due to study design [41,42]. The recruitment of HS cases from a tertiary centre could skew towards patients with severe disease presenting to specialist care and a potential underrepresentation of patients with mild disease who are treated in the community. Additionally, the potential effects that the clinical management of patients with HS has on their metabolic health outcome (or vice versa) was not assessed. 

## 5. Conclusions

In conclusion, the results of this study suggest that the assessment of metabolic risk is an essential component in the clinical management of both obesity and HS. Dermatologists caring for patients with HS may be in a position of identifying individuals who are at increased risk of cardiometabolic pathology therefore allowing for the timely institution of appropriate risk management strategies.

## Figures and Tables

**Table 1:** Salient characteristics of the study population. Quantitative variables are presented as medians (interquartile range). HOMA-IR = Homeostasis Model Assessment-Insulin resistance, ALP = Alkaline phosphatase, GGT = Ɣ-glutamyl transferase, ALT = alanine transferase, LDL= low density lipoprotein.

	Controls (*n* = 521)	HS (*n* = 111)	*p* Value
N (%) Female	330 (63.3%)	54 (48.6%)	<0.01
N (%) Smokers/Ex-smokers	165 (31.7%)	71 (64%)	<0.01
Metabolic Syndrome (NCEP-ATPIII)	88 (20.3%)	36 (32.4%)	<0.001
N (%) Type 2 Diabetes	23 (4.6%)	8 (7.2%)	0.178
N (%) Hurley I	-	53 (47.7%)	
N (%) Hurley II	-	41 (36.9%)	
N (%) Hurley III	-	17 (15.3%)	
Age years	41 (6)	34 (25)	<0.001
Body Mass Index kg/m^2^	27.5 (7.8)	31 (10.8)	<0.001
Waist Circumference cm	89 (20)	100 (25)	<0.001
Systolic Blood Pressure mmHg	120 (10)	133 (23)	<0.001
Diastolic Blood pressure mmHg	80 (10)	79.5 (16)	0.488
Fasting plasma glucose mmol/L	5.13 (0.66)	5.06 (0.9)	0.472
HOMA-IR	1.65 (1.17)	1.83 (1.24)	0.031
HbA1c *%*	5.3 (0.4)	5.3 (0.6)	0.126
ALP U/L	63 (21)	72 (33)	<0.001
GGT U/L	19 (16)	23 (22)	0.05
ALT U/L	17 (13)	16 (12)	0.185
Total Cholesterol mmol/L	4.87 (1.1)	4.73 (1.66)	0.749
LDL-C mmol/L	2.85 (1.06)	3 (1.43)	0.326
HDL-C mmol/L	1.41 (0.49)	1.27 (0.52)	<0.001
Triglycerides mmol/L	1.01 (0.71)	1.11 (0.73)	0.067
Visceral Adiposity Index	1.09 (1.1)	1.31 (1.49)	0.01
Atherogenic Index of Plasma	−0.16 (0.42)	−0.08 (0.45)	0.013
Platelet lymphocyte ratio	134.39 (62.25)	135.29 (73.5)	0.604
Neutrophil lymphocyte ratio	2.02 (0.89)	2.34 (0.99)	0.001
Red cell distribution width %	14.62 (2.48)	15.91 (2.77)	<0.001

**Table 2:** Clinical and biochemical characteristics of the HS and control groups stratified into the four different body composition phenotypes. Metabolic health defined by NCEP-ATPIII criteria. Quantitative variables are presented as medians (interquartile range) WC–waist circumference, SBP–systolic blood pressure, DBP–Diastolic blood pressure, FPG–fasting plasma glucose, ALP–Alkaline phosphatase, GGT–Ɣ glutamyl transferase, ALT–Alanine transaminase, TC–Total cholesterol, LDL–Low density lipoprotein, HDL–high density lipoprotein, TG–triglycerides, VAI–visceral adiposity index, AIP–Atherogenic index of plasma, PLR–platelet-lymphocyte ratio, NLR–Neutrophil lymphocyte ratio.

	METABOLIC HEALTH AS DEFINED BY HOMA-IR								
	Metabolically Healthy Normal Weight			Metabolically Healthy Overweight Obese			Metabolically Unhealthy Overweight Obese		
	Control Group (*n* = 147)	HS Group (*n* = 20)	*p* Value	Control Group (*n* = 269)	HS Group (*n* = 61)	*p* Value	Control Group (*n* = 86)	HS Group *(n* = 27)	*p* Value
Sex (% Female)	118 (80)	11 (55)	**0.011**	154 (57.2)	30 (49.2)	0.252	42 (48.5)	13 (48.1)	0.95
Smoking status (%Ex/Smoker)	49 (33)	12 (60)	**0.02**	76 (28.3)	38 (62.3)	**0.01**	34 (39.5)	19 (70.4)	**0.005**
Age years	41 (7)	31 (20.5)	**0.001**	41 (6)	30 (25)	**<0.001**	42 (6)	39 (20)	0.228
BMI kg/m^2^	22.4 (2.7)	23.05 (1.4)	0.164	29.1 (5.7)	31 (8.8)	**0.033**	32.55 (7.1)	39.1 (9.5)	**<0.001**
WC cm	74 (11)	78.5 (8.5)	0.053	92 (15)	100 (18)	**0.001**	102 (17)	115 (23)	**<0.001**
SBP mmHg	120 (15)	125 (16)	**0.005**	120 (10)	132 (22)	**<0.001**	120 (15)	140 (20)	**<0.001**
DBP mmHg	80 (10)	73 (10)	**0.03**	80 (5)	79 (14)	0.53	80 (5)	86 (15)	**0.004**
FPG mmol/L	4.94 (0.560)	4.89 (0.71)	0.971	5.13 (0.58)	4.99 (0.86)	0.078	5.69 (1.61)	5.58 (2.59)	0.664
HOMA-IR	1.12 (0.85)	1.55 (1.02)	**0.012**	1.64 (0.84)	1.61 (0.72)	0.316	3.07 (0.97)	3.25 (1.37)	0.21
Hba1c %	5.2 (0.3)	5.2 (0.55)	0.439	5.3 (0.49)	5.3 (0.6)	0.568	5.6 (1.3)	5.5 (1.5)	0.933
ALP	56 (20)	64.5 (31)	**0.007**	65 (19)	72 (27)	0.002	70.5 (23)	77 (33)	0.471
GGT	14 (9)	15 (14)	0.29	20 (18)	22 (17)	0.938	28.5 (22)	34 (38)	0.259
ALT	14 (9)	13.5 (7.5)	0.303	19 (14)	17 (12)	**0.041**	23 (17)	22 (16)	0.424
TC	4.64 (1.14)	4.59 (1.52)	0.79	4.86 (1.03)	4.77 (1.61)	0.706	5.06 (1.33)	4.73 (2.18)	0.147
LDL-C	2.62 (1.07)	2.61 (1.49)	0.517	2.89 (1.01)	3.01 (1.21)	0.343	3.14 (1.24)	2.92 (2.09)	0.505
HDL-C	1.63 (0.5)	1.36 (0.62)	**0.019**	1.39 (0.39)	1.32 (0.48)	**0.04**	1.1 (0.31)	1.11 (0.4)	0.979
TG	0.77 (0.38)	0.94 (0.39)	**0.026**	1.07 (0.64)	1.11 (0.84)	0.389	1.72 (1.11)	1.25 (0.81)	**0.046**
VAI	0.75 (0.51)	1.01 (1.12)	**0.033**	1.11 (0.93)	1.24 (1.55)	0.143	2.48 (2.04)	1.89 (1.66)	0.064
AIP	−0.32 (0.26)	−0.11 (0.32)	**0.001**	−0.14 (0.35)	−0.15 (0.48)	0.378	0.2 (0.33)	0.04 (0.37)	0.051
PLR	142.17 (53.64)	145.71 (55.22)	0.212	129.9 (57.6)	130.37 (52.68)	0.905	125.22 (57.89)	135.5 (80.5)	0.554
NLR	2.06 (0.99)	2.33 (0.73)	0.078	1.96 (0.89)	2.23 (0.97)	**0.014**	2.07 (0.81)	2.43 (1.17)	**0.061**

**Table 3:** Clinical and biochemical characteristics of the HS and control groups stratified into the four different body composition phenotypes. Metabolic health defined by NCEP-ATPIII criteria. Quantitative variables are presented as medians (interquartile range) WC–waist circumference, SBP–systolic blood pressure, DBP–Diastolic blood pressure, FBG–fasting plasma glucose, ALP–Alkaline phosphatase, GGT–Ɣ glutamyl transferase, ALT–Alanine transaminase, TC–Total cholesterol, LDL–Low density lipoprotein, HDL–high density lipoprotein, TG–triglycerides, VAI–visceral adiposity index, AIP–Atherogenic index of plasma, PLR–platelet-lymphocyte ratio, NLR–Neutrophil lymphocyte ratio.

	METABOLIC HEALTH AS DEFINED NCEP-ATPIII								
	Metabolically Healthy Normal Weight			Metabolically Healthy Overweight Obese			Metabolically Unhealthy Overweight Obese		
	Control Group (*n* = 131)	HS Group (*n* = 16)	*p* Value	Control Group (*n* = 217)	HS Group (*n* = 47)	*p* Value	Control Group (*n* = 148)	HS Group (*n* = 42)	*p* Value
Sex(% Female)	109 (83.2)	8 (50)	**0.02**	144 (66.4)	25 (53.2)	**0.088**	59 (39.9%)	18 (42.9%)	0.727
Smoking status (%Ex/Smoker)	45 (34.4)	8 (50%)	0.218	62 (28.6)	25 (53.2%)	**0.01**	50 (33.8%)	33 (78.6%)	**<0.001**
Age years	41 (7)	30 (14)	**<0.001**	40 (6)	28 (18)	**<0.001**	42 (5.5)	40.5 (24)	0.858
BMI kg/m^2^	22.5 (2.5)	22.7 (1.65)	0.377	29 (6.1)	31.3 (7.7)	0.101	31.1 (6.95)	36.7 (11.5)	**<0.001**
WC cm	74 (10)	79 (12)	**0.031**	91.4 (15)	95 (15)	**0.024**	100 (17.75)	113 (20)	**<0.001**
SBP mmHg	115 (15)	124 (13)	**0.011**	120 (10)	128 (23)	**<0.001**	122 (14)	140 (20)	**<0.001**
DBP mmHg	80 (10)	71.5 (11)	**0.005**	80 (10)	79 (14)	0.454	80 (5)	85 (17)	**0.012**
FBG mmol/L	4.92 (0.49)	4.89 (0.59)	0.818	5.06 (0.47)	4.88 (0.60)	**0.004**	5.69 (0.83)	5.72 (1.3)	0.746
HOMAIR	1.13 (0.91)	1.57 (0.86)	**0.014**	1.69 (1.04)	1.61 (0.73)	0.416	2.21 (1.32)	2.49 (1.52)	0.22
Hba1c %	5.2 (0.4)	5.25 (0.55)	0.172	5.2 (0.4)	5.1 (0.4)	0.16	5.5 (0.55)	5.8 (1)	**0.027**
ALP	55 (20)	61 (28)	0.135	64 (17)	74 (30)	**0.002**	69 (26)	72 (30)	0.239
GGT	14 (8)	17 (14)	0.113	18 (13)	18 (16)	0.887	28.5 (25.5)	32 (24)	0.473
ALT	14 (9)	13.5 (7.5)	0.501	16 (13)	14 (11)	0.125	24 (19)	22.5 (16)	0.176
TC	4.58 (1.22)	4.76 (1.13)	0.576	4.79 (0.89)	4.46 (1.59)	0.206	5.1 (1.53)	5.35 (1.98)	0.905
LDL-C	2.6 (0.98)	2.61 (1.39)	0.285	2.84 (0.94)	2.71 (1.08)	0.657	3.15 (1.29)	3.39 (1.62)	0.481
HDL-C	1.66 (0.52)	1.42 (0.58)	0.073	1.41 (0.46)	1.4 (0.44)	0.423	1.27 (0.38)	1.11 (0.39)	**<0.001**
TG	0.75 (0.4)	0.99 (0.37)	**0.028**	0.97 (0.59)	0.87 (0.55)	0.411	1.53 (0.97)	1.65 (1.4)	0.385
VAI	0.74 (0.51)	1.01 (0.71)	0.059	1.06 (0.78)	1.01 (0.81)	0.819	1.84 (1.63)	2.36 (2.13)	0.076
AIP	−0.35 (0.27)	−0.09 (0.3)	**0.003**	−0.17 (0.35)	−0.18 (0.34)	0.765	0.11 (0.37)	0.18 (0.45)	0.895
PLR	142.17 (55.57)	161.86 (51.47)	0.067	134.39 (57.15)	134.07 (49.83)	0.922	123.58 (62)	127.6 (79.79)	0.802
NLR	2.06 (0.95)	2.3 (0.63)	0.332	1.99 (0.84)	2.43 (0.89)	**0.011**	1.97 (0.93)	2.29 (1.32)	0.054

## Data Availability

Data is available from the corresponding author upon request.

## References

[1] Martora F., Megna M., Battista T., Potestio L., Annunziata M.C., Marasca C., Villani A., Fabbrocini G. (2023). Adalimumab, Ustekinumab, and Secukinumab in the Management of Hidradenitis Suppurativa: A Review of the Real-Life Experience. Clin. Cosmet. Investig. Dermatol..

[2] Mintoff D., Benhadou F., Pace N.P., Frew J.W. (2021). Metabolic syndrome and hidradenitis suppurativa: Epidemiological, molecular, and therapeutic aspects. Int. J. Dermatol..

[3] González-López M.A., Vilanova I., Ocejo-Viñals G., Arlegui R., Navarro I., Guiral S., Mata C., Pérez-Paredes M.G., Portilla V., Corrales A. (2020). Circulating levels of adiponectin, leptin, resistin and visfatin in non-diabetics patients with hidradenitis suppurativa. Arch. Dermatol. Res..

[4] Mintoff D., Agius R., Benhadou F., Das A., Frew J.W., Pace N.P. (2023). Obesity and Hidradenitis Suppurativa: Targeting meta-inflammation for therapeutic gain?. Clin. Exp. Dermatol..

[5] Wellen K.E., Hotamisligil G.S. (2005). Inflammation, stress, and diabetes. J. Clin. Investig..

[6] Jais A., Brüning J.C. (2017). Hypothalamic inflammation in obesity and metabolic disease. J. Clin. Investig..

[7] Elías-López D., Vargas-Vázquez A., Mehta R., Bautista I.C., Olvera F.D.R., Gómez-Velasco D., Valdes P.A., Aguilar-Salinas C.A., Metabolic Syndrome Study Group (2021). Natural course of metabolically healthy phenotype and risk of developing Cardiometabolic diseases: A three years follow-up study. BMC Endocr. Disord..

[8] Tsatsoulis A., Paschou S.A. (2020). Metabolically Healthy Obesity: Criteria, Epidemiology, Controversies, and Consequences. Curr. Obes. Rep..

[9] Eckel N., Mühlenbruch K., Meidtner K., Boeing H., Stefan N., Schulze M.B. (2015). Characterization of metabolically unhealthy normal-weight individuals: Risk factors and their associations with type 2 diabetes. Metabolism.

[10] Commodore-Mensah Y., Lazo M., Tang O., Echouffo-Tcheugui J.B., Ndumele C.E., Nambi V., Wang D., Ballantyne C., Selvin E. (2021). High Burden of Subclinical and Cardiovascular Disease Risk in Adults with Metabolically Healthy Obesity: The Atherosclerosis Risk in Communities (ARIC) Study. Diabetes Care.

[11] Opio J., Croker E., Odongo G.S., Attia J., Wynne K., McEvoy M. (2020). Metabolically healthy overweight/obesity are associated with increased risk of cardiovascular disease in adults, even in the absence of metabolic risk factors: A systematic review and meta-analysis of prospective cohort studies. Obes. Rev..

[12] Stefan N., Schulze M.B. (2023). Metabolic health and cardiometabolic risk clusters: Implications for prediction, prevention, and treatment. Lancet Diabetes Endocrinol..

[13] Jørgensen A.H.R., Yao Y., Ghazanfar M.N., Ring H.C., Thomsen S.F. (2020). Burden, predictors and temporal relationships of comorbidities in patients with hidradenitis suppurativa: A hospital-based cohort study. J. Eur. Acad. Dermatol. Venereol..

[14] Egeberg A., Gislason G.H., Hansen P.R. (2016). Risk of Major Adverse Cardiovascular Events and All-Cause Mortality in Patients with Hidradenitis Suppurativa. JAMA Dermatol..

[15] Garg A., Malviya N., Strunk A., Wright S., Alavi A., Alhusayen R., Alikhan A., Daveluy S.D., Delorme I., Goldfarb N. (2022). Comorbidity screening in hidradenitis suppurativa: Evidence-based recommendations from the US and Canadian Hidradenitis Suppurativa Foundations. J. Am. Acad. Dermatol..

[16] Hambly R., Kearney N., Hughes R., Fletcher J.M., Kirby B. (2023). Metformin Treatment of Hidradenitis Suppurativa: Effect on Metabolic Parameters, Inflammation, Cardiovascular Risk Biomarkers, and Immune Mediators. Int. J. Mol. Sci..

[17] Mintoff D., Camilleri L., Aquilina S., Boffa M.J., Clark E., Scerri L. (2020). Prevalence of hidradenitis suppurativa in Malta: Comparison with established epidemiological data. Clin. Exp. Dermatol..

[18] Ralf Paus L., Kurzen H., Kurokawa I., Jemec G.B.E., Emtestam L., Sellheyer K., Giamarellos-Bourboulis E.J., Nagy I., Bechara F.G., Sartorius K. (2008). What causes hidradenitis suppurativa?. Exp. Dermatol..

[19] Hurley H. (1989). Axillary hyperhidrosis, apocrine bromhidrosis, hidradenitis suppurativa, and familial benign pemphigus: Surgical approach. Dermatol. Surg. Princ. Pract..

[20] Canoui-Poitrine F., Le Thuaut A., Revuz J.E., Viallette C., Gabison G., Poli F., Pouget F., Wolkenstein P., Bastuji-Garin S. (2013). Identification of three hidradenitis suppurativa phenotypes: Latent class analysis of a cross-sectional study. J. Investig. Dermatol..

[21] Agius R., Pace N.P., Fava S. (2021). Characterisation of body size phenotypes in a middle-aged Maltese population. J. Nutr. Sci..

[22] Matthews D.R., Hosker J.P., Rudenski A.S., Naylor B.A., Treacher D.F., Turner R.C. (1985). Homeostasis model assessment: Insulin resistance and beta-cell function from fasting plasma glucose and insulin concentrations in man. Diabetologia.

[23] Kuk J.L., Ardern C.I. (2009). Are metabolically normal but obese individuals at lower risk for all-cause mortality?. Diabetes Care.

[24] Durward C.M., Hartman T.J., Nickols-Richardson S.M. (2012). All-cause mortality risk of metabolically healthy obese individuals in NHANES III. J. Obes..

[25] Hinnouho G.-M., Czernichow S., Dugravot A., Nabi H., Brunner E.J., Kivimaki M., Singh-Manoux A. (2015). Metabolically healthy obesity and the risk of cardiovascular disease and type 2 diabetes: The Whitehall II cohort study. Eur. Heart J..

[26] Bo S., Musso G., Gambino R., Villois P., Gentile L., Durazzo M., Cavallo-Perin P., Cassader M. (2012). Prognostic implications for insulin-sensitive and insulin-resistant normal-weight and obese individuals from a population-based cohort. Am. J. Clin. Nutr..

[27] Amato M.C., Giordano C., Galia M., Criscimanna A., Vitabile S., Midiri M., Galluzzo A. (2010). Visceral Adiposity Index. Diabetes Care.

[28] Dobiásová M., Frohlich J. (2001). The plasma parameter log (TG/HDL-C) as an atherogenic index: Correlation with lipoprotein particle size and esterification rate in apoB-lipoprotein-depleted plasma (FER(HDL)). Clin. Biochem..

[29] Witte-Händel E., Wolk K., Tsaousi A., Irmer M.L., Mößner R., Shomroni O., Lingner T., Witte K., Kunkel D., Salinas G. (2019). The IL-1 Pathway Is Hyperactive in Hidradenitis Suppurativa and Contributes to Skin Infiltration and Destruction. J. Investig. Dermatol..

[30] Shlyankevich J., Chen A.J., Kim G.E., Kimball A.B. (2014). Hidradenitis suppurativa is a systemic disease with substantial comorbidity burden: A chart-verified case-control analysis. J. Am. Acad. Dermatol..

[31] De Vita V., Fabbrocini G. (2018). Mechanical Stress as a Cause of Hidradenitis Suppurativa: A Lesson from a Patient with a Monster Hernia. Acta Dermatovenerol. Croat. ADC.

[32] Monfrecola G., Balato A., Caiazzo G., De Vita V., Di Caprio R., Donnarumma M., Lembo S., Fabbrocini G. (2016). Mammalian target of rapamycin, insulin resistance and hidradenitis suppurativa: A possible metabolic loop. J. Eur. Acad. Dermatol. Venereol..

[33] Ruze R., Liu T., Zou X., Song J., Chen Y., Xu R., Yin X., Xu Q. (2023). Obesity and type 2 diabetes mellitus: Connections in epidemiology, pathogenesis, and treatments. Front. Endocrinol..

[34] Agamia N.F., Sorror O.A., Sayed N.M., Ghazala R.A., Echy S.M., Moussa D.H., Melnik B.C. (2023). Overexpression of hypoxia-inducible factor-1α in hidradenitis suppurativa: The link between deviated immunity and metabolism. Arch. Dermatol. Res..

[35] Abu Rached N., Gambichler T., Ocker L., Dietrich J.W., Quast D.R., Sieger C., Seifert C., Scheel C., Bechara F.G. (2023). Screening for Diabetes Mellitus in Patients with Hidradenitis Suppurativa-A Monocentric Study in Germany. Int. J. Mol. Sci..

[36] Ingram J.R. (2020). The epidemiology of hidradenitis suppurativa. Br. J. Dermatol..

[37] Ahlqvist E., Storm P., Käräjämäki A., Martinell M., Dorkhan M., Carlsson A., Vikman P., Prasad R.B., Aly D.M., Almgren P. (2018). Novel subgroups of adult-onset diabetes and their association with outcomes: A data-driven cluster analysis of six variables. Lancet Diabetes Endocrinol..

[38] González-López M.A., Hernández J.L., Lacalle M., Mata C., López-Escobar M., López-Mejías R., Portilla V., Fuentevilla P., Corrales A., González-Vela M.C. (2016). Increased prevalence of subclinical atherosclerosis in patients with hidradenitis suppurativa (HS). J. Am. Acad. Dermatol..

[39] Oba M.C., Askin O., Gunver M.G., Kocaarslan G., Alis D.C., Engin B. (2023). Subclinical atherosclerosis in patients with hidradenitis suppurativa treated with TNF inhibitors. Skin Res. Technol..

[40] Sánchez-Díaz M., Salvador-Rodríguez L., Cuenca-Barrales C., Arias-Santiago S., Molina-Leyva A. (2023). Potential Predictors of Cardiovascular Risk Improvement in Patients with Hidradenitis Suppurativa Treated with Adalimumab: A Pivotal Study of Factors Associated with Carotid Intima-Media Thickness Reduction. Dermatol. Ther..

[41] Eckel N., Li Y., Kuxhaus O., Stefan N., Hu F.B., Schulze M.B. (2018). Transition from metabolic healthy to unhealthy phenotypes and association with cardiovascular disease risk across BMI categories in 90 257 women (the Nurses’ Health Study): 30 year follow-up from a prospective cohort study. Lancet Diabetes Endocrinol..

[42] Kabat G.C., Wu W.Y.-Y., Bea J.W., Chen C., Qi L., Stefanick M.L., Chlebowski R.T., Lane D.S., Wactawski-Wende J., Wassertheil-Smoller S. (2017). Metabolic phenotypes of obesity: Frequency, correlates and change over time in a cohort of postmenopausal women. Int. J. Obes..

